# Carotid body, insulin, and metabolic diseases: unraveling the links

**DOI:** 10.3389/fphys.2014.00418

**Published:** 2014-10-29

**Authors:** Sílvia V. Conde, Joana F. Sacramento, Maria P. Guarino, Constancio Gonzalez, Ana Obeso, Lucilia N. Diogo, Emilia C. Monteiro, Maria J. Ribeiro

**Affiliations:** ^1^CEDOC, Centro Estudos Doenças Crónicas, NOVA Medical School, Faculdade de Ciências Médicas, Universidade Nova de LisboaLisboa, Portugal; ^2^Health Research Unit - UIS, School of Health Sciences, Polytechnic Institute of LeiriaLeiria, Portugal; ^3^Departamento de Bioquímica y Biología Molecular y Fisiología, Facultad de Medicina, Instituto de Biología y Genética Molecular, Consejo Superior de Investigaciones Científicas, Ciber de Enfermedades Respiratorias, CIBERES, Instituto de Salud Carlos III, Universidad de ValladolidValladolid, España

**Keywords:** carotid body, chronic intermittent hypoxia, insulin resistance, metabolic dysfunction, obstructive sleep apnea

## Abstract

The carotid bodies (CB) are peripheral chemoreceptors that sense changes in arterial blood O_2_, CO_2_, and pH levels. Hypoxia, hypercapnia, and acidosis activate the CB, which respond by increasing the action potential frequency in their sensory nerve, the carotid sinus nerve (CSN). CSN activity is integrated in the brain stem to induce a panoply of cardiorespiratory reflexes aimed, primarily, to normalize the altered blood gases, via hyperventilation, and to regulate blood pressure and cardiac performance, via sympathetic nervous system (SNS) activation. Besides its role in the cardiorespiratory control the CB has been proposed as a metabolic sensor implicated in the control of energy homeostasis and, more recently, in the regulation of whole body insulin sensitivity. Hypercaloric diets cause CB overactivation in rats, which seems to be at the origin of the development of insulin resistance and hypertension, core features of metabolic syndrome and type 2 diabetes. Consistent with this notion, CB sensory denervation prevents metabolic and hemodynamic alterations in hypercaloric feed animal. Obstructive sleep apnea (OSA) is another chronic disorder characterized by increased CB activity and intimately related with several metabolic and cardiovascular abnormalities. In this manuscript we review in a concise manner the putative pathways linking CB chemoreceptors deregulation with the pathogenesis of insulin resistance and arterial hypertension. Also, the link between chronic intermittent hypoxia (CIH) and insulin resistance is discussed. Then, a final section is devoted to debate strategies to reduce CB activity and its use for prevention and therapeutics of metabolic diseases with an emphasis on new exciting research in the modulation of bioelectronic signals, likely to be central in the future.

## The carotid bodies

**Figure 1:**
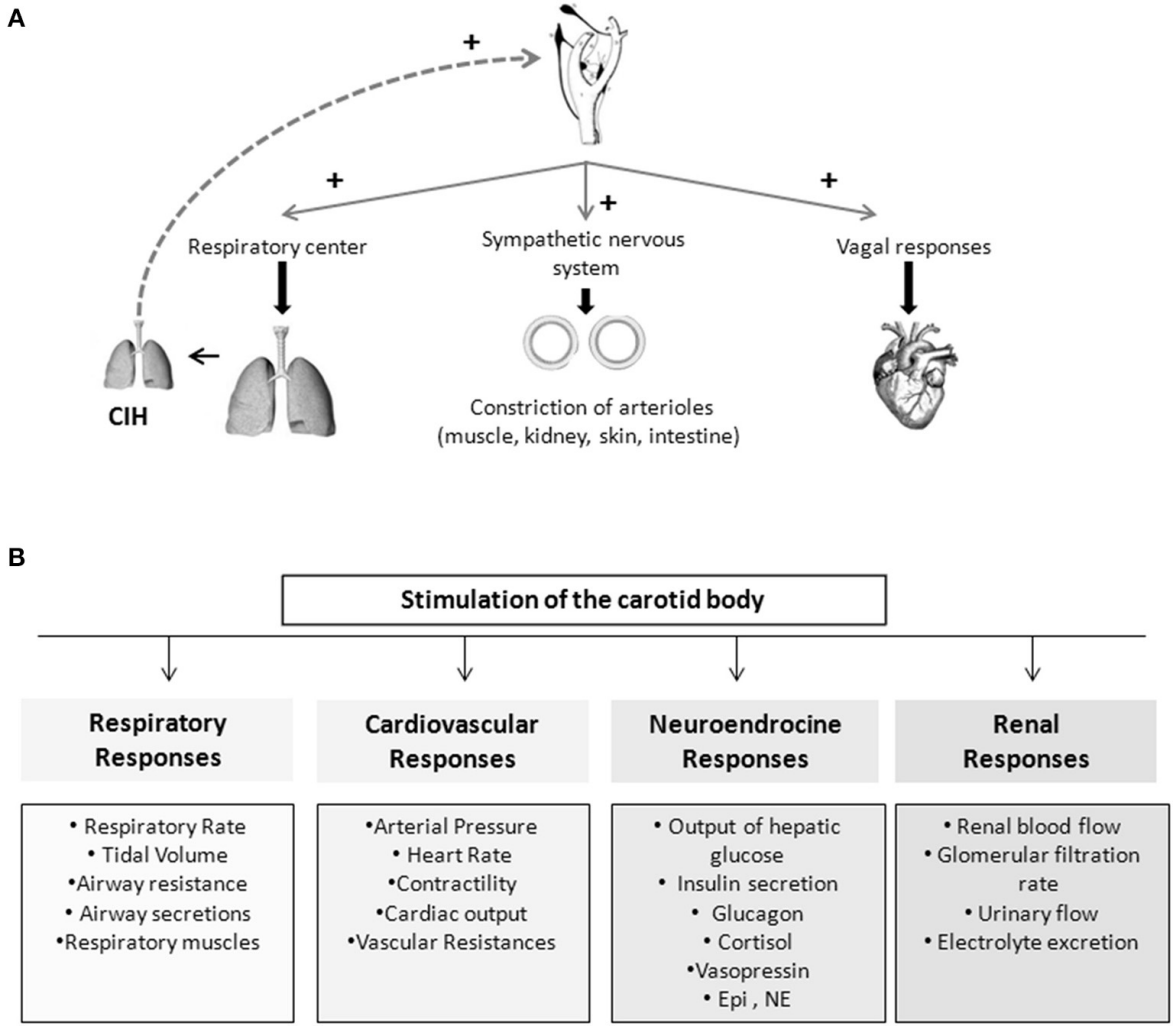
**Schematic representation of the chemoreflexes elicited by the carotid bodies. (A)** Representation of important mechanism involved in the reflex-responses elicited by the carotid body. **(B)** Stimulation of the carotid body is capable of produce cardiovascular, respiratory, endocrine, and renal responses.

The carotid bodies (CB) are peripheral chemoreceptors located bilaterally in the bifurcation of the common carotid artery that classically sense changes in arterial blood such as low O_2_ (hypoxia), high CO_2_ (hypercapnia), and low pH (acidosis). Hypoxia and acidosis/hypercapnia activate the CB, inducing an increase in the frequency of discharge in the nerve endings of its sensorial nerve, the carotid sinus nerve (CSN). The CSN activity is integrated in the *nucleus solitary tract* to induce a myriad of respiratory reflexes aimed to normalize the altered blood gases, via hyperventilation (Gonzalez et al., 1994), and to regulate blood pressure and cardiac performance via an increase in the activity of the sympathetic branch of the autonomic nervous system (SNS) (Marshall, 1994) (see Figure 1). The chemoreceptor cells, also known as glomus or type I cells, are the main cellular constituent of the CB and are generally accepted as its chemosensory unit. These cells, which are derived of the neural crest, contain several classical neurotransmitters including, catecholamines [CA; dopamine (DA), and norepinephrine (NE)], serotonin, ACh, neuropeptides (substance P and enkephalins) and adenosine (Ado) and ATP (Gonzalez et al., 1994; Zhang et al., 2000; Rong et al., 2003; Buttigieg and Nurse, 2004; Conde and Monteiro, 2004; Conde et al., 2012a). All these substances, their agonists and antagonists are capable of modifying, inhibiting or stimulating CSN activity. In addition to chemoreceptor cells, the CB also possesses type II cells, or sustentacular cells and it has been proposed that they are adult neural stem cells sustaining neurogenesis *in vivo* in response to physiological stimuli, like chronic hypoxia, and acting in paracrine signaling during hypoxia (Pardal et al., 2007; Piskuric and Nurse, 2013).

## Role of carotid body in metabolism

### Evidences for a role of carotid body in glucose homeostasis

The idea of a physiological role of the CB on the control of glucose metabolism was first suggested by Petropavlovskaya in the 50's. In this pioneer study it was shown that the stimulation of the CB induces a reflex hyperglycemia, an effect that is mediated by the adrenal medulla, since it was not observed in adrenalectomized animals (Petropavlovskaya, 1953). Twenty five years later, Alvarez-Buylla and de Alvarez-Buylla (1988) confirmed those results by demonstrating that the pharmacological stimulation of the CB with cyanide (NaCN) produced an increase in hepatic glucose output in cats, this reflex response being eliminated by bilateral adrenalectomy or by surgical removal of the neurohypophysis (Alvarez-Buylla et al., 1997). Also, it was shown that changes in blood concentration in the CB-CSN, superfused *in vivo*, modify brain glucose retention, suggesting that chemosensory activity in the CSN controls brain glucose metabolism (Alvarez-Buylla and de Alvarez-Buylla, 1994). In parallel with the increase in hepatic glucose output, one would expect an increase in plasma insulin levels to ensure an adequate glucose utilization by the peripheral tissues and, in fact, stimulation of CBs by corconium, a nicotinomimetic agent, caused a rise in circulating insulin that was reversed by CSN resection (Anichkov and Tomilina, 1962). Later on, Koyama et al. (2000) demonstrated that CB plays an important role in glucose homeostasis *in vivo*, since dogs that have their CB resected presented lower arterial glucagon in basal conditions and reduced glucagon and cortisol levels during insulin-induced hypoglycemia, together with a marked decrease in endogenous hepatic glucose production in response to hypoglycemia, and with an increase in insulin sensitivity, independent of blood glucose level. These last results suggested for the first time that CB resection affects the response to moderate hyperinsulinemia and therefore, that the CB may play a role in glucose homeostasis that is not related with the hypoglycemic counterregulatory response.

The results obtained by Koyama et al. (2000) were supported by clinical studies where it was demonstrated that, the rate of glucose infusion necessary to maintain glucose levels in a hyperinsulinemic-hypoglycemic clamp was significantly higher during hyperoxia than in normoxia (Wehrwein et al., 2010). In the same study, the authors also observed that hyperoxia, which blunts CB activity, decreased the release of counter-regulatory hormones such as adrenaline, cortisol, glucagon and growth hormone, which seems to indicate that the CB play an important role in neuroendocrine responses during hypoglycemia (Wehrwein et al., 2010). However, the absence of adequate controls in hyperinsulinemic-euglycemic conditions in this study does not allow assigning the effects to the hyperinsulinemia *per se* or to hypoglycemia. In another clinical study designed to determine whether hypo- and hyperglycaemia modulate the ventilatory responses to hypoxia, it was shown that hypoglycemia, as well as hyperglycemia, produced an increase in ventilation and in the hypoxic ventilatory response, being the latter accompanied by an increase in circulating counter-regulatory hormones (Ward et al., 2007). Interestingly, both hypo- and hyperglycemia were obtained under hyperinsulinemic conditions, and therefore it is possible that the effect in ventilation observed was due to hyperinsulinemia rather than to altered glucose concentrations. More recently, our laboratory has shown that CBs are overactivated in diet-induced animal models of insulin resistance and hypertension (Ribeiro et al., 2013). Also, we have demonstrated that insulin resistance and hypertension produced by hypercaloric diets are completely prevented by chronic bilateral CSN resection, and these results strengthen the link between CB dysfunction and the development of insulin resistance (Ribeiro et al., 2013). In addition, we observed that CSN resection in control animals decreased insulin sensitivity, suggesting that CB also contributes to maintain metabolic control in physiological conditions (Ribeiro et al., 2013). Therefore, the research in the field performed since Petropavlovskaya work in the early 1950's strongly supports that the CB is a key organ in glucose homeostasis and that its dysfunction contributes to the pathogenesis of metabolic disturbances.

### Glucose sensing in the carotid body

One of the hypotheses that came out to explain the role of the CB in glucose homeostasis was the potential of the CB as a glucosensor. Whereas some *in vivo* and *in vitro* studies, performed in cultured CB chemoreceptor cells or slices, had shown that CB could respond to blood glucose levels, (Koyama et al., 2000; Pardal and Lopez-Barneo, 2002; Zhang et al., 2007) others have completely denied a direct involvement of the CB in glucose sensing (Almaraz et al., 1984; Bin-Jaliah et al., 2004, 2005; Conde et al., 2007; Fitzgerald et al., 2009; Gallego-Martin et al., 2012). Due to these controversial results, the sensitivity of the CB to hypoglycaemia is still a hot topic in the CB field.

In cultured CB slices, perfusion with low or glucose-free solutions at a PO_2_ ≈150 mmHg produced an increase in CAs release from chemoreceptor cells with a magnitude comparable to the response evoked by hypoxia and potentiated hypoxic responses (Pardal and Lopez-Barneo, 2002). Moreover it was found that low glucose inhibited K^+^ currents (Pardal and Lopez-Barneo, 2002) in an extent similar to the observed by Peers during intense hypoxia (Peers, 1990); low glucose also promoted Ca^2+^ entry in chemoreceptor cells (Pardal and Lopez-Barneo, 2002). Lopez-Barneo's group published that sensitivity to low glucose and to hypoxia depends on different signal transduction mechanisms, although they converge on the final steps causing transmembrane Ca^2+^ influx and transmitter release (García-Fernández et al., 2007). Almost at the same time, but using an experimental model of co-culture of type I clusters and afferent petrosal neurons, Zhang et al. (2007) described that low glucose increased the spiking activity in the neurons, this increase being sensitive to purinergic and nicotinic blockers, implying that low glucose stimulates chemoreceptor cells and promotes the release of ATP and ACh. Contrasting with these results, CSN activity in freshly isolated cat and rat CB–CSN preparation was not modified by perfusion with glucose-free or low-glucose solutions (Almaraz et al., 1984; Bin-Jaliah et al., 2004, 2005). Also, Conde et al. (2007) demonstrated that low glucose concentrations neither activate the release of neurotransmitters, namely CAs and ATP, from the CB, nor altered basal and hypoxia (5% O_2_)-induced CSN action potential frequency in freshly isolated whole CB preparations (Conde et al., 2007). In the same line, Fitzgerald et al. (2009) showed that the release of ATP from the cat CB was not modified in the presence of hypoglycemia but, surprisingly, they observed an increase in the release of ACh in the same conditions (Fitzgerald et al., 2009). Additionally, it was shown that withdrawal of glucose from the perfusion media did not activate the K_ATP_ channels, suggesting that this channel was insensitive to hypoglycemia (Kim et al., 2011). Altogether these results suggest that low glucose is not a direct stimulus for the CB chemoreceptors and do not support a significant physiological role of the CB as a glucose sensor.

Several differences can account for these discrepant results regarding glucose sensing in the CB, namely species differences, different dissociation protocols or culture conditions that lead to an altered cells phenotype, as suggested by Kumar (2007), or even the differences in the PO_2_ levels used by some authors, as postulated by Zhang et al. (2007). However, Conde et al. (2007) have shown in the whole CB that low or absent glucose does not activate either chemoreceptor cells or the CB–CSN complex at different PO_2_ tested in a very wide range (~133, 66, 46, and 33 mmHg) and thus, differences in the PO_2_ used in the experiments in intact preparations vs. slices or co-cultures is not the factor determining divergent findings, as suggested by Zhang et al. (2007). More recently, Gallego-Martin et al. (2012) demonstrated that in intact CBs cultured during 1 day, but not in freshly isolated organs, 0 mM glucose media potentiates the release of CAs elicited by hypoxia and that chemoreceptor cells in culture become transiently more dependent on glycolysis suggesting that the scarcity of glucose leads the cells to acquire the ability to increase their neurosecretory response to hypoxia. Another relevant issue in the discussion is the duration of glucose deprivation. While glucose reduction or deprivation did not have an effect when applied for short periods of time (<15 min), either in basal conditions or in response to hypoxia, when applied for longer periods of time (up to 120 min) it caused a spontaneous increase in basal release of CAs observable after 40 min of glucose deprivation. Concomitantly, bursts of CSN activity were observed with a comparable time course to the release of CAs, that culminated in a complete loss of the capacity of the CSN to respond to hypoxia (Conde et al., 2007). Consistent with these findings Holmes et al. (2014) have recently demonstrated that basal CSN activity was sustained during glucose deprivation approximately for 30 min before irreversible failure following a brief period of increased activity. Also, they showed that pharmacological inhibition of glycogenolysis and depletion of glycogen reduced the time to glycolytic run down, suggesting that glycogen metabolism in chemoreceptor cells allows glycogenolysis and the maintenance of CSN basal activity during hypoglycemia (Holmes et al., 2014). Therefore, glycogen metabolism may account for the differences reported in the capacity of the CB to sense glycemia and could contribute to CB responses in pathological conditions associated with an overstimulation of the organ.

### Is insulin a stimulus for CB activation?

**Figure 2:**
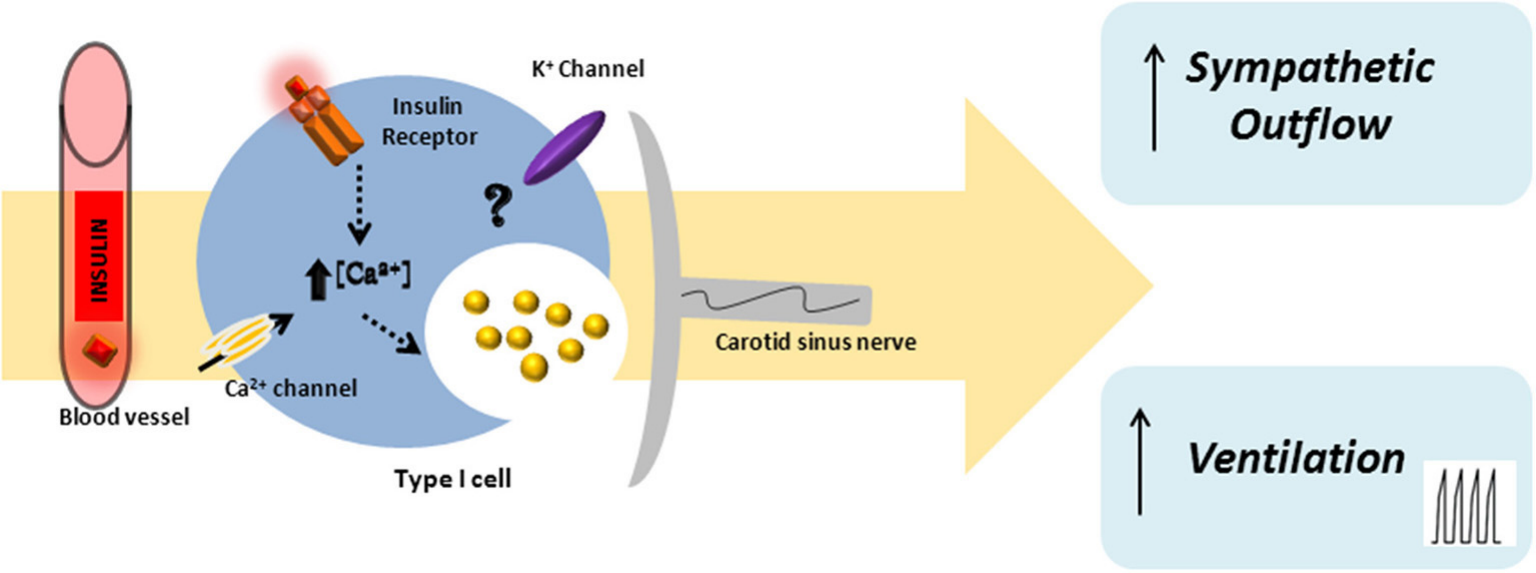
**Schematic representation of insulin action in the carotid body**. Insulin will act on insulin receptors present in the carotid body chemoreceptor cells eliciting an increase in intracellular Ca^2+^ and the release of neurotransmitters, such as dopamine and ATP. The insulin-induced neurosecretory response in chemoreceptor cells is transduced in an increase in ventilation and in an augmented sympathetic outflow.

**Figure 3:**
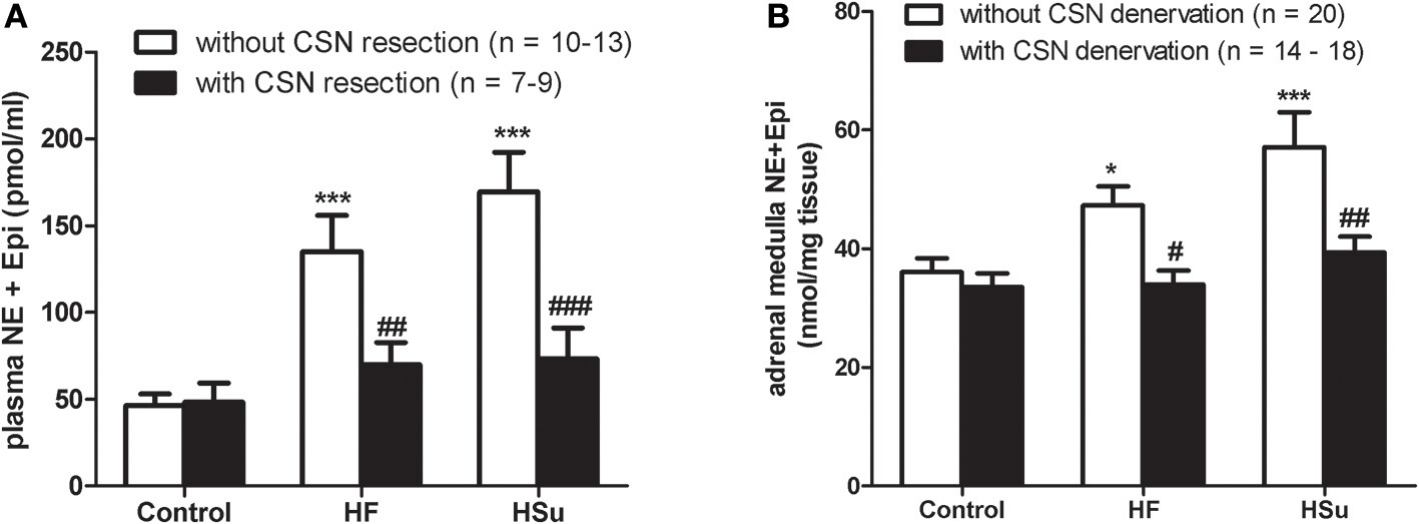
**Effect of carotid sinus nerve resection on sympathetic nervous system activity, measured as circulating catecholamines [norepinephrine (NE) + epinephrine (Epi)] (A) and adrenal medulla catecholamines (NE + Epi) content (B), in control, high fat (HF) and high sucrose (HSu) diet rats**. Bars represent mean ± s.e.m. Two-Way ANOVA with Bonferroni multicomparison tests; ^*^p < 0.05, ^***^p < 0.001 vs. control; ^#^*p* < 0.05, ^##^*p* < 0.01, ^###^*p* < 0.001 vs. values within the same group (adapted from Ribeiro et al., 2013).

**Figure 4:**
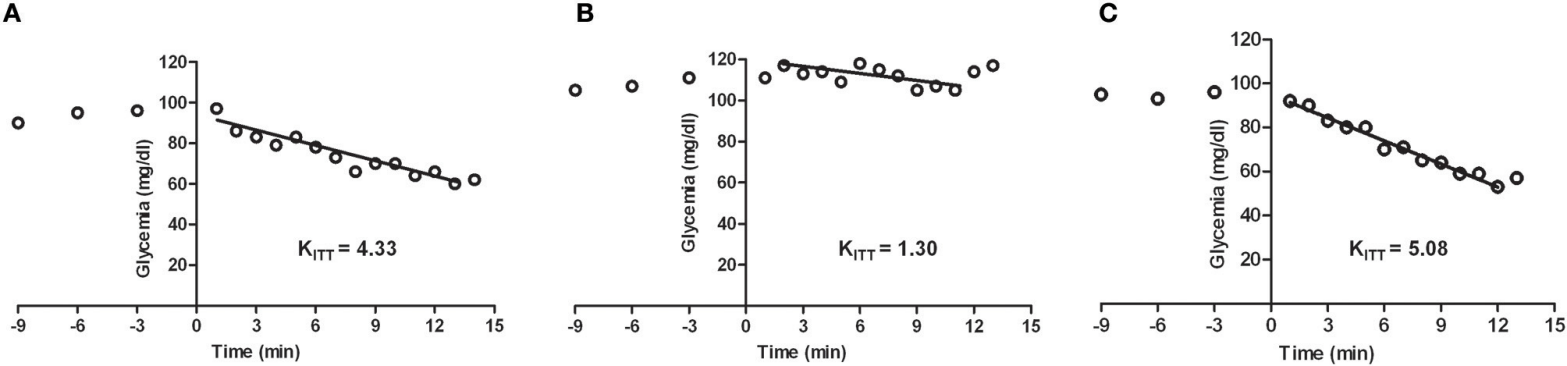
**Representative excursion curves for the insulin tolerance test in control (A), high fat (HF) (B), and high fat animals submitted to carotid sinus nerve resection (C) rats**. Note that insulin sensitivity, expressed by the constant of the insulin tolerance test (KITT) decrease in the HF animals, this decrease being completely prevented by the bilateral resection of the carotid sinus nerve. HF animals were achieved by submitting the animals to a HF diet (45% lipid-rich diet) during 21 days. Bilateral resection of the carotid sinus nerve **(C)** was performed 5 days prior to submitting the animals to HF diet (adapted from Ribeiro et al., 2013).

A large body of literature supports a role for the central nervous system in insulin-induced sympathoexcitation, as the injection of insulin on *arcuate nucleus* and *paraventricular nucleus* has been shown to produce an increase in spinal sympathetic outflow, mediated by dorsal hypothalamus and rostral ventrolateral medulla (for a review see Dampney, 2011). However, this effect cannot be exclusively assigned to a centrally-mediated mechanism, since the injection of insulin into the carotid artery of anesthetized dogs produces an increase in blood pressure and sympathetic activity higher than the systemic insulin administration, being the effect abolished by ganglionic blockade (Pereda et al., 1962). These results were the first to suggest a role for the peripheral nervous system in insulin-mediated sympathetic activity. During the evaluation of a putative direct role of the CB in glucose sensing, Bin-Jaliah et al. (2004) observed that insulin infusion, used to produce hypoglycemia, increased minute ventilation and the rate of O_2_ consumption (VO_2_), an effect that was totally mediated by the CB, since CSN denervation blunted it. The same authors demonstrated afterwards that insulin-induced hypoglycemia was associated with a significantly increase in CO_2_ chemosensitivity, an effect that was mediated by the CB, since the effect was lost in animals that had their CSN resected (Bin-Jaliah et al., 2005). Since *in vitro* hypoglycemia was incapable of modifying basal CSN activity (Bin-Jaliah et al., 2004; Conde et al., 2007) and blunted the response of CSN to hypercapnia (Bin-Jaliah et al., 2005) the elevation of ventilation observed *in vivo* by Bin-Jaliah's group was somehow surprising (Bin-Jaliah et al., 2004, 2005) and the hypothesis of being an indirect consequence of systemic hypoglycemia related to some other undetermined substance had to be considered. To pursue this hypothesis, our group has been dedicated to investigate whether insulin itself is capable of stimulating the CB and of eliciting a neurosecretory response. We have demonstrated the presence of insulin receptors in the rat CB by western-blot and its phosphorylation in response to insulin (Ribeiro et al., 2013). The presence of insulin receptors was also confirmed on finding that isolated whole CBs incubated with insulin accumulate more 2-deoxiglucose than the diaphragm muscle (Gallego Martin et al., 2014). Insulin is also capable to induce a rise in intracellular Ca^2+^ in chemoreceptor cells and to elicit the release of ATP and dopamine from the whole CB in a concentration-dependent manner (Ribeiro et al., 2013). As schematically represented in Figure 2, we have also shown that this neurosecretory response is transduced into an increase in ventilation in the whole animal, as insulin increased the spontaneous ventilation in a dose-dependent manner during an euglycemic clamp (Ribeiro et al., 2013). The increase in ventilation induced by insulin is mediated by the CB, since it is absent in animals that had their CSN resected (Ribeiro et al., 2013). Contrarily to our results, Bin-Jaliah et al. (2004) proposed that the ventilatory and metabolic effects observed *in vivo* were not due to insulin *per se*, since the increase in ventilation produced by insulin was absent during an euglycemic clamp. However, some differences in the methodology used can be in the basis of these discrepancies. In our study we have administrated a bolus of insulin intracarotidally to guarantee that the first site of insulin action is the CB, and not systemically as Bin-Jaliah et al. (2004, 2005). Also we performed a dose-response curve in which several concentrations of insulin were tested, making the results more robust in terms of concluding on a role of insulin in CB modulation. In fact, the neurosecretory response and the increase in ventilation elicited by insulin in our experimental setting support the idea that insulin is a very powerful stimulus for CB activation. Nevertheless, these findings do not exclude that the central nervous system is also involved in the sympathetic activation observed in response to circulating insulin and more studies are required to clarify the exact contribution of both the peripheral and the central nervous system in this process. It is undoubtedly however, that the overactivation of the SNS, measured as the increase in plasmatic CAs (norepinephrine + epinephrine) and in CAs (norepinephrine + epinephrine) content of the adrenal medulla (Figure 3) and the insulin resistance (Figure 4) seen in hypercaloric animal models are prevented by surgical resection of the CSN. These findings point toward a new role for the CB in the regulation of peripheral insulin sensitivity and in the pathogenesis of insulin resistance (Ribeiro et al., 2013).

## Linking insulin, sympathetic nervous system activation and metabolic dysfunction: the role of the carotid body

The sympathetic nervous system (SNS) is an important component of the autonomic nervous system playing a major role in the maintenance of homeostasis due to its involvement in the control of the cardiovascular system and of several metabolic processes. Sympathetic overactivity has been associated with several diseases, such as cardiovascular diseases (Graham et al., 2004), kidney disease (Converse et al., 1992), and metabolic disturbances, including type 2 diabetes (Huggett et al., 2003; Grassi et al., 2005, 2007; Kobayashi et al., 2010). In metabolic diseases the increase in sympathetic activation has been attentively associated with hyperinsulinemia, hyperleptinemia increased non-esterified free fatty acids, inflammation, and obesity among others, however the precise mechanisms remain to be unequivocally elucidated (Lambert et al., 2010).

### Insulin-induced sympathetic overactivation

It is known since the early 80's that insulin stimulates sympathetic nerve activity (Rowe et al., 1981) and, more recently, it has been shown that this stimulation occurs at blood insulin concentrations within the physiological range (Hausberg et al., 1995). In fact, the relationship between hyperinsulinemia and the increased sympathetic nerve activity lead Landsberg to propose in 1986 a causal relationship between metabolic disturbances, such as insulin resistance and dyslipidemia, and overactivation of the SNS (Landsberg, 1986).

In the last decades several reports were published, both in animals and in humans, supporting the hypothesis that insulin increases sympathetic nerve activity. In humans insulin has been shown to increase muscle sympathetic nerve activity (MSNA) (Anderson et al., 1991; Scherrer et al., 1993; Vollenweider et al., 1993) as well as norepinephrine levels (Anderson et al., 1991; Lambert et al., 2010) in euglycemic conditions. The MSNA response observed in response to insulin administration is both gradual (Anderson et al., 1991; Scherrer et al., 1993; Vollenweider et al., 1993, 1994; Banks, 2004) and sustained because MSNA remains increased even after plasma insulin levels return to baseline (Anderson et al., 1991; Scherrer et al., 1993; Vollenweider et al., 1993, 1994; Banks, 2004). In rats and dogs, insulin infusion also increases sympathetic nerve activity along with an increase in plasma norepinephrine levels (Liang et al., 1982; Tomiyama et al., 1992). However, the discovery that insulin infusion did not increase sympathetic nerve activity in the skin in humans (Berne et al., 1992) and also that graded increases in plasma insulin failed to significantly increase renal or adrenal sympathetic activity in rats though leading to increased lumbar SNS activity, lead to the hypothesis that hyperinsulinemia produces regionally non-uniform increases in sympathetic nerve activity (Morgan et al., 1993). Also, while some authors claim that the relationship between insulin concentrations and sympathetic nerve activity is dose-dependent (Anderson et al., 1991; Berne et al., 1992), others have shown that this relationship is not apparent (Vollenweider et al., 1993, 1994) attributing this effect to a saturation of the receptors needed for insulin to cross the blood brain barrier (Banks et al., 1997; Dampney, 2011). The slow rise and fall in MSNA produced by hyperinsulinemia would be explained by the time insulin needs to cross the blood brain barrier (Banks, 2004).

As reviewed previously, our group demonstrated that insulin is capable of stimulating the CB eliciting a hyperventilatory response (Ribeiro et al., 2013) (Figure 2). These results are in accordance with the recent findings by Limberg et al. (2014) where hyperoxic silencing of carotid chemoreceptors reduced MSNA in hyperinsulinemic conditions, suggesting that the CB also mediates insulin-dependent sympathoexcitation in humans (Limberg et al., 2014).

### The role of carotid body in metabolic dysfunction

**Figure 5:**
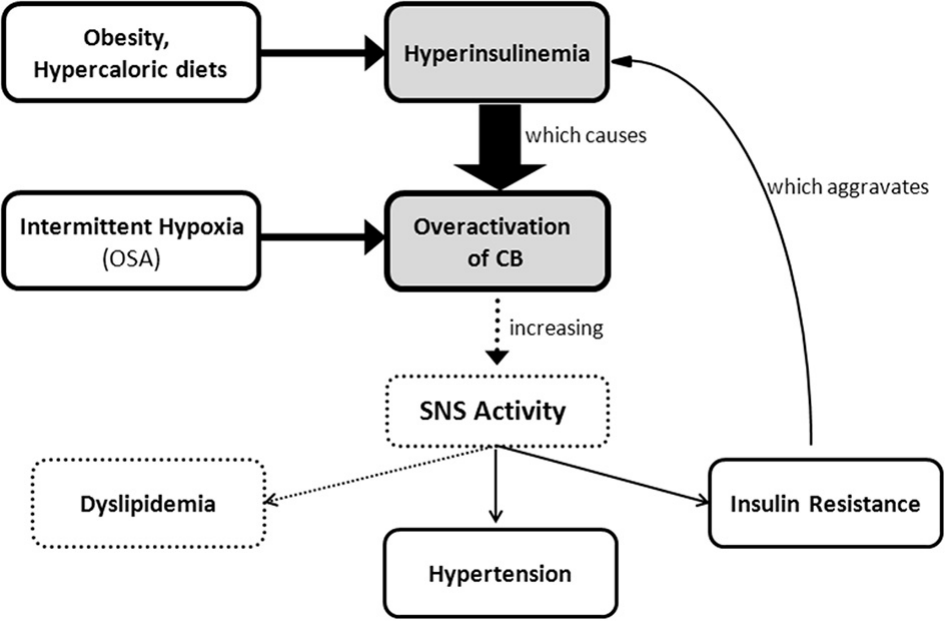
**Schematic representation of carotid body involvement in the development of insulin resistance through an increase in sympathetic nervous system activity**. Overactivation of the carotid body caused by hyperinsulinemia and/or by chronic intermittent hypoxia originates an increase in sympathetic nervous system activity that promotes insulin resistance, hypertension, and probably dyslipidemia.

SNS activation is implicated in the pathogenesis of metabolic diseases and in the specific components of the metabolic syndrome, such as insulin resistance, hypertension, dyslipidemia and obesity (Kahn and Flier, 2000; Esler et al., 2006; Tentolouris et al., 2006; Mancia et al., 2007). The idea that sympathetic hyperactivity contributes to the development of insulin resistance is not new (Defronzo, 1981), although the mechanisms involved in the association between sympathetic nerve activity and insulin resistance (Egan, 2003; Tentolouris et al., 2006; Tsioufis et al., 2007, 2011), are complex and not clearly understood, and several questions remain unanswered, including how is promoted the sustained activation of the SNS that characterizes metabolic diseases. Our group has recently proposed that the CB is the common link between sympathetic nerve activity, insulin resistance and hypertension (Ribeiro et al., 2013) (Figure 5). The CBs contribute to regulate blood pressure and cardiac performance via SNS activation (Marshall, 1994) and through an increased sympathetic drive, the CB directly activates the adrenals and increases the sympathetic vasoconstrictor outflow to muscle, splanchnic, and renal beds (Marshall, 1994; Cao and Morrison, 2001; Schultz et al., 2007). Therefore, we have hypothesized that an overactivation of the CB contributes to the genesis of insulin resistance, core pathological feature of metabolic disorders as type 2 diabetes or the metabolic syndrome. In fact, we have shown that animal models of diet-induced prediabetes develop an overactivation of the CB; measured as an increased spontaneous ventilation as well as increased respiratory responses to ischemic hypoxia; increased hypoxia-evoked release of dopamine and increased expression of tyrosine hydroxilase (Ribeiro et al., 2013). This overactivation of the CB results in an increase in SNS activity, measured as circulating CAs and the adrenal medulla CAs content (Figure 3), and in an reduction in insulin sensitivity (Figure 4) (Ribeiro et al., 2013). All these characteristic features of metabolic diseases were prevented by CSN resection (Ribeiro et al., 2013) meaning that the CB is primordial in controlling peripheral insulin sensitivity and that CB dysfunction is involved in the genesis of these disturbances.

## Linking obstructive sleep apnea with metabolic dysfunction

### Obstructive sleep apnea

Obstructive sleep apnea (OSA) is the most common form of sleep disorder. It is characterized by repetitive collapse of the pharyngeal airway during sleep, which generally requires arousal to re-establish airway patency and resume breathing (Pillar and Shehadeh, 2008). Upper airway obstruction can result in either absent (apneas) or reduced (hypopneas) ventilation (Dempsey et al., 2010), despite persisting respiratory efforts, such that ventilatory requirements are not met. Consequently, hypoxemia and hypercapnia develop, which further stimulate respiratory effort. However, without spontaneous airway opening, the increased drive is ineffective to increase ventilation. Therefore, the apnea/hypopnea typically continues until the patient arouses from sleep and ends the obstruction. Following airway re-opening, hyperventilation occurs to reverse the blood gas disturbances that developed during the respiratory event. The patient then returns to sleep and another obstruction develops (Eckert et al., 2009). The repetitive nature of these events results in the excessive daytime sleepiness (Punjabi et al., 1999), fatigue and neurocognitive dysfunction (Kim et al., 1997). Patients with OSA are classically characterized by the apnea-hypopnea index in mild OSA (≥5 and <15 events/hour), moderate OSA (≥15 and <30 events/hour), and severe OSA (≥30 events/hour) (Kapur, 2010). OSA of at least mild severity (five or more events per hour of sleep) affects 5–20% of the general population (Young et al., 1993, 2002) with a prevalence of 17–24% in men and 5–9% in women, and a tendency to even out after the menopause (Young et al., 1993; Bixler et al., 1998, 2001). The higher risk factors associated with OSA are age, male gender, and high body mass index. and this sleep disturbance is also linked to increased risk of hypertension, insulin resistance, glucose intolerance, type 2 diabetes, dyslipidemia, atherosclerosis and non-alcoholic fatty liver disease (Nieto et al., 2000; Newman et al., 2001; Punjabi et al., 2004; Drager et al., 2005; Reichmuth et al., 2005; Pulixi et al., 2014). The most effective and well-studied treatment for OSA is continuous positive airway pressure (CPAP) devices, which maintain upper airway patency during sleep, promote sleep continuity and significantly improve subjective and objective measures of daytime sleepiness (Patel et al., 2003).

The association between OSA and hypertension is well established (see Wolf et al., 2010 for a review). Bixler et al. (2000) demonstrated that OSA was independently associated with hypertension, both in men and women, being this relationship strongest in young subjects and proportional to the severity of the disease. The underlying mechanisms of OSA-induced hypertension are not completely understood, however it has been demonstrated that sympathetic activation plays a central role in the pathophysiological process. OSA patients, exhibit elevated blood pressure and elevated muscle sympathetic tone, as well as increased plasma CAs, an effect that diminishes with CPAP treatment (Somers et al., 1995; Kara et al., 2003). This high sympathetic drive is present even during daytime wakefulness when subjects are breathing normally and both arterial oxygen saturation and carbon dioxide levels are also normal (Kara et al., 2003; Narkiewicz and Somers, 2003). It was suggested that intermittent hypoxia resulting from apneas is the primary stimulus for evoking sympathetic excitation (Prabhakar et al., 2007, 2012) and that hypercapnia that occurs during apneas and even apnea, by itself, also contribute to sympathetic excitation (Prabhakar and Kumar, 2010; but see Lesske et al., 1997). Since the CB is the primary sensor for hypoxia and the ensuing reflex activates sympathetic nerve activity and elevates blood pressure (Lesske et al., 1997; Prabhakar and Kumar, 2010), it was suggested that CB overactivation by CIH produced by apneas would result in an increased sympathetic activity and hypertension. In fact, the surgical denervation of the CB prevented the increase in mean arterial blood pressure induced by CIH, as well as the adrenal demedullation and the chemical denervation of the peripheral SNS by 6-hydroxy dopamine (Lesske et al., 1997). The involvement of an increased sympatho-adrenal tone in CIH induced-hypertension was also suggested by the finding that acute hypoxia in CIH animals evoked the release of CAs from *ex vivo* adrenal medulla, an effect that is absent in controls, suggesting that direct activation adrenal medulla may account for the increase in blood pressure and plasma CAs seen in CIH animals (Kumar et al., 2006). In addition to the sympathetic tone, endothelial dysfunction, oxidative stress and inflammation have been proposed as potential mechanisms involved in the onset of the hypertension (see Gonzalez et al., 2012). However, evidence for a unique pathogenic mechanism has been difficult to establish in OSA patients because of concomitant co morbidities (Iturriaga et al., 2009; Del Rio et al., 2012).

### Chronic intermittent hypoxia: linking carotid body and obstructive sleep apnea

Chronic intermittent hypoxia (CIH), characterized by cyclic hypoxic episodes of short duration followed by normoxia, is a characteristic feature of OSA. The CB has been proposed to mediate the reflex increase in sympathetic activity and blood pressure associated with OSA due to CIH (Narkiewicz et al., 1999). In fact, several studies have demonstrated an increase in peripheral CB drive in OSA subjects. This increased CB peripheral drive was reflected by enhanced ventilatory and cardiovascular reflex responses induced by acute hypoxia (Somers et al., 1995; Narkiewicz et al., 1999) and also by an increase in basal tidal volume (Loredo et al., 2001). In a pioneer study, Fletcher et al. (1992a) demonstrated that 5 weeks of CIH induced an elevation of blood pressure in rats both during exposure to hypoxia and subsequently. In a succeeding publication, the same authors described that bilateral CB denervation prevented the development of hypertension in rats exposed to CIH for 35 days (Fletcher et al., 1992b), indicating that CB chemoreceptors are fundamental for the progression of CIH induced-hypertension. Consistent with these findings it was also demonstrated that CB denervation prevented the CIH-induced sympathetic activation (Prabhakar et al., 2005). In the last decade several reports have strengthened the idea that CIH resulting from sleep-disordered breathing leads to an overactivation of the CB, manifested by its increased sensitivity to hypoxia (Rey et al., 2004; Prabhakar et al., 2007; Peng et al., 2009). The recording of CSN discharge *in vitro* and *in situ* showed that exposure of animals to CIH increases the basal CSN discharge and enhances the chemosensory response to acute hypoxia (Peng et al., 2003; Rey et al., 2004; Gonzalez-Martín et al., 2011). Furthermore, Peng et al. (2003) demonstrated that CIH induces a CSN chemosensory long-term facilitation characterized by progressive increase in CSN activity with each hypoxic episode, remaining the baseline activity elevated approximately during 60 min after the last acute hypoxic stimuli. These authors have also suggested that, since the increase in CB sensory activity triggers sympathetic nerve discharge and an increase in blood pressure, sensory long-term facilitation contributes to the persistent increase in SNA and blood pressure that is observed in recurrent apnea patients (Peng et al., 2003). Peng et al. (2003) also found that when CIH-exposed rats were re-exposed to normoxia, the long-term facilitation and the augmented hypoxic ventilatory response was reversed. The reversible nature of the CB responses to CIH might explain why CPAP therapy reverses the adverse cardio-sympathetic effects in OSA patients (Kara et al., 2003). Also, CIH has no significant effect on CB weight (Obeso et al., 2012) nor morphology, as CIH did not produce significant differences in the total volume of the CB, number of glomus cells or glomus cell volume (Peng et al., 2003). The mechanisms underlying the CB overactivation induced by CIH are not well understood, with this effect being attributed to increased levels of endothelin-1 (Rey et al., 2006) and to reactive oxygen species (ROS) in the CB (Peng et al., 2003, 2009); however local expression of chemosensory modulators, like nitric oxide, and pro-inflammatory cytokines in the CB may have different temporal contribution to the CB chemosensory potentiation induced by CIH (Prabhakar et al., 2005; Del Rio et al., 2011). Nevertheless, the possibility that alterations in the storing capacity and dynamics of possibly several neurotransmitter systems (e.g., CAs) (Gonzalez-Martín et al., 2011) cannot be excluded and changes in the density and/or affinity of their receptors in the sensory nerve endings could account for the overactivation of the CB seen in CIH.

### Obstructive sleep apnea, chronic intermittent hypoxia, and metabolic dysfunction

It is now consensual that OSA is independently associated with metabolic syndrome, which incorporates visceral obesity, hypertension, glucose intolerance, insulin resistance, and dyslipidemia (Bonsignore et al., 2013). Several studies have reported that metabolic syndrome is highly prevalent in OSA patients, with rates between 50 and >80% (Bonsignore et al., 2013). The indication of a relationship between OSA and the various pathological features of the metabolic syndrome, particularly insulin resistance, is recent when compared with the considerable body of evidence indicating that OSA can independently contribute to the development of sustained daytime hypertension. One of the earliest studies that showed that OSA is independently associated with insulin resistance was the performed by Ip et al. (2002), where the degree of insulin resistance was matched with body mass index and severity of OSA among 185 patients. Through a multiple linear regression, the authors found that obesity was the primary determinant of insulin resistance, but the patient's apnea-hypopnea index and minimal arterial O_2_ saturation were also significantly contributors (Ip et al., 2002). In 2004 a large epidemiological study directly assessed OSA prevalence by polysomnography and measured glucose and insulin levels under fasting and after an oral glucose tolerance test in a subset of 2656 subjects from the Sleep Heart Health Study. The authors showed that subjects with mild or moderate to severe OSA had elevated fasting glucose and impaired oral glucose tolerance (Punjabi et al., 2004). Also, they demonstrated that the effect of OSA on glucose intolerance was independently associated with age, gender, body mass index and waist circumference (Punjabi et al., 2004). In another study, Punjabi and Beamer (2009), performed an intravenous glucose tolerance test in 118 non-diabetic subjects and found that the apnea-hypopnea index and the severity of nocturnal oxyhemoglobin desaturation were associated with decreased insulin sensitivity and pancreatic β-cell dysfunction, the effect being independent of age, sex and percent body fat (Punjabi and Beamer, 2009).

As expected by its association with insulin resistance, OSA may also be a risk factor for the development of type 2 diabetes, according to two large prospective studies. These two studies showed that regular snoring is associated with a 2- to 7-fold risk for type 2 diabetes over a period of 10 years (Elmasry et al., 2000; Al-Delaimy et al., 2002). Since snoring is not a clinical diagnostic for OSA, in a longitudinal study, Reichmuth et al. (2005) analyzed the data from 1387 subjects in the Wisconsin Sleep Cohort and examining the association between OSA, diagnosed by polysomnography, and the development of type 2 diabetes. Comparable to previous cross-sectional studies, a positive association between clinically diagnosed OSA and type 2 diabetes, after adjustment for age, sex, and waist girth was shown (Reichmuth et al., 2005). However, in a follow-up study of 978 subjects, the odds ratio for developing type 2 diabetes within a 4 years period for those with an apnea-hypopnea index of >15 events/hour did not reach statistical significance after adjustment for waist girth (Reichmuth et al., 2005). Since it is well described that insulin resistance precedes in approximately 10–15 years the development of type 2 diabetes (Nathan, 2002), the limitation of this work may be related with the duration of follow-up that was only 4 years. Therefore, further longitudinal studies would be necessary to fully examine the role of OSA in the development of type 2 diabetes.

The link between OSA and metabolic dysfunction was also sustained by the results obtained by Babu et al. (2005) showing that CPAP treatment for 3 months decreased postprandial glucose levels and glycated hemoglobin in type 2 diabetes patients with OSA, being the decrease higher when CPAP was used for more than 4 h per night (Babu et al., 2005). Also, Harsch et al. (2004a) observed an increase in insulin sensitivity, assessed through a hyperinsulinemic-euglycemic clamp, in type 2 diabetes patients after 3 months of effective CPAP treatment. In another study performed by Harsch et al. (2004b), in OSA patients without type 2 diabetes, it was observed that CPAP treatment increased insulin sensitivity within 2 days of therapy, with further improvements occurring at the 3 months follow-up. In contrast with the reported beneficial effects of CPAP on glucose metabolism and insulin resistance in OSA patients, some studies demonstrated that CPAP treatment for 3 or 6 months did not improve fasting glucose or insulin plasma levels (Ip et al., 2000). These differences among studies may be related with the treatment duration, lack of a control group, insufficient statistical power and absence of data on CPAP compliance.

The exact mechanism for the pathological changes that occur in glucose metabolism and insulin action in OSA patients is not completely understood. It is possible that multiple interrelated factors contribute to the complex interactions between OSA, obesity and glucose control. OSA is intrinsically associated with CIH and sleep loss due to sleep fragmentation, and both induce insulin resistance (Tasali et al., 2008). Recently, a lot of research has been published devoted to the study CIH and metabolic dysfunction in rodents however some of the data obtained is not consensual. It has been shown that mice exposed during 30 days to CIH exhibited elevated levels of fasting plasma insulin but comparable glucose levels and higher homeostasis model assessment (HOMA) index, indicating insulin resistance, an effect that was attributed to a pancreatic β-cell dysfunction (Wang et al., 2013). These results were sustained by the recent work of Gonzalez group where they observe that 15 days of CIH in rats induce insulin resistance, assessed by the HOMA index without affecting fasting glucose plasma levels and glucose tolerance (Olea et al., 2014). These findings obtained in mice and rats contrast with the recent publication by Shin and co-workers where they show that 4/6 weeks of CIH in mice increased fasting blood glucose, baseline hepatic glucose output but not insulin sensitivity measured through a hyperinsulinemic euglycemic clamp (Shin et al., 2014). These effects being mediated by the CB as CSN denervation prevented the CIH-induced hyperglycemia and the increase in hepatic glucose output (Shin et al., 2014). Whereas the differences obtained in several metabolic parameters, like fasting glycemia, can be due to distinct species studied as well as to the different CIH paradigms, we must refer that HOMA index is a human index, an must not be used as the only index to assess insulin resistance in rodents.

Several intermediate mechanisms have been proposed to explain the pathological alterations in glucose metabolism in OSA: increased sympathetic activation, deregulation of the hypothalamus-pituitary axis and generation of ROS (Tasali et al., 2008). In addition, pancreatic β-cells are highly sensitive to hypoxia, and the subsequent shift to anaerobic glycolytic metabolism favors insulin resistance (Pallayova et al., 2011). Also, it was recently shown that mice exposed to 30 days CIH exhibited pancreatic β-cell dysfunction, manifested by impaired glucose-stimulated insulin secretion and increased mitochondrial ROS (Wang et al., 2013), which may contribute to the development of type 2 diabetes among sleep apnea patients. Finally, the oxidative status and activation of inflammatory pathways can also contribute to deregulation of metabolism (Tasali et al., 2008). It has been recently shown that 15 days to CIH in rats induce an oxidative status manifested by an increase in lipid peroxides and diminished activities of superoxide dismutases, an inflammatory status characterized by augmented C-reactive protein and nuclear factor kappa-B activation and a sympathetic hyperactivity assessed by plasma and renal artery CA levels and synthesis rate (Olea et al., 2014). Also, the same authors have shown that, as expected, the combination of CIH and obesity worsened the alterations observed (Olea et al., 2014).

Obesity is considered a major risk factor for the development and progression of OSA. It is estimated that 40% of obese individuals have OSA; consequently approximately 70% of individuals with OSA are obese (Vgontzas et al., 2000; Daltro et al., 2007). One possible mechanisms by which obesity may worsen OSA is due to fat deposition at specific sites of the body, namely in the upper airways. In fact, fat deposition in the tissues surrounding the upper airway appears to result in a smaller lumen and increased collapsibility of the upper airway, predisposing to apnea (Shelton et al., 1998; Schwab et al., 2003). This increase in fat deposition next to the upper airways can be found even in non-obese subjects with OSA (Mortimore et al., 1998). Fat deposits around the thorax (truncal obesity) also reduce chest compliance and functional residual capacity, and may increase oxygen demand (Naimark and Cherniack, 1960). Another fat depot that can contribute to OSA is visceral fat. Visceral obesity is common in subjects with OSA and is closely related with an increase in apnea index (Shinohara et al., 1997), Since obesity is positively correlated with OSA, weight loss and weight gain prevention offer a successful therapeutic approach to reduce the occurrence and the severity of OSA and its related mortality. In a longitudinal study, Peppard et al. (2000) showed that a 10% of weight loss predicted a 26% decrease in the apnea-hypopnea index, which suggest that even a modest weight loss may be effective in managing OSA and reducing new occurrence of OSA. Furthermore, CPAP treatment for 6 months led to visceral fat loss even if subjects did not lose weight (Chin et al., 1999). Short sleep fragmentation is associated with decreased levels of leptin, a hormone that lowers food intake, increases energy expenditure (Friedman and Halaas, 1998) and is secreted in proportion to body fat stores (Considine et al., 1996). In OSA subjects, several studies reported increased leptin levels compared to weight-matched control (Ip et al., 2000; Vgontzas et al., 2000), which correlated with OSA severity (Ip et al., 2000), and decreased after CPAP treatment (Chin et al., 1999).

Although obesity is the primary risk factor for OSA this disease also affects lean subjects, as Pamidi et al. (2012) demonstrated that young lean men, free of cardiometabolic disease, the presence of OSA is associated with IR and compensatory hyperinsulinemia to maintain normal glucose homeostasis (Pamidi et al., 2012). Therefore, from this study we can conclude that OSA may increase the risk of type 2 diabetes independently of traditional cardiometabolic risk factors. In the Sleep Heart Study (Seicean et al., 2008), a large community-based cohort of older individuals (>65 years of age), the presence of OSA was associated with a higher prevalence of prediabetes and occulted type 2 diabetes in the non-overweight group. Furthermore, the effect of CPAP treatment may be different between obese and non-obese subjects. Harsch et al. (2004b) showed that the improvement in insulin sensitivity was much smaller in obese subjects than in non-obese subjects, suggesting that in obese individual's insulin sensitivity is mainly determined by obesity and, to a smaller extent, by sleep apnea.

Obesity is known to be strongly associated with metabolic dysfunction, and that contributes to insulin resistance and glucose intolerance (Landsberg, 1996, 2001), nevertheless metabolic dysfunction can be present in lean OSA subjects (Pamidi et al., 2012). In CIH rodent models metabolic dysfunction is present without the obesity component (Carreras et al., 2012; Fenik et al., 2012; Wang et al., 2013; Shin et al., 2014), as it was described that animals submitted to CIH gain less weight (Carreras et al., 2012) or the similar weight (Olea et al., 2014) in comparison with controls. Also, the amounts of perirenal and epididymal fat found in CIH animals was similar to those found in controls (Olea et al., 2014). Taken together these results show that in OSA, obesity is not the only factor that contributes to metabolic dysfunction. The involvement of CB has been recently proposed as one of the links between CIH and sympathetic overactivity and metabolic dysfunction, since CB denervation prevents CIH-induced fasting hyperglycemia, although CB denervation was incapable of prevent insulin resistance (Shin et al., 2014), suggesting that other mechanisms can account for the CIH induced-insulin resistance. In fact, little is known regarding the molecular mechanisms behind this relationship, with the reduction of Glut4 metabolic fraction in skeletal muscle in CIH animals being the only mechanism described (Carreras et al., 2012). Therefore, detailed studies on the molecular mechanisms of insulin action in insulin-sensitive tissues will contribute enormously to better understand the paradigm of CIH-induced insulin resistance, and so the relationship between OSA and metabolic dysfunction.

## Future perspectives

In the last couple of years, several reports of non-classical roles of the CB on glucose homeostasis and metabolic regulation have been published, contributing to launch the CB as a putative therapeutic target for the treatment of endocrine diseases. Our group has been actively involved in the process and recently we described that chronic CB overstimulation is implicated in the etiology of diet-induced insulin resistance (Ribeiro et al., 2013). We have also described that surgical resection of the CSN prevents the development of dysmetabolic changes induced by hypercaloric treatments in rats (Ribeiro et al., 2013), an observation that contributed to strengthen that CB blockade/modulation represents a novel and unexploited therapeutic approach.

Besides the surgical resection of the CB, its overactivation can also be prevented pharmacologically with an old, well-studied and very safe drug: caffeine. Sustained caffeine administration prevents the development of hypertension, impaired glucose tolerance and insulin resistance in prediabetes animal models (Conde et al., 2012b; Panchal et al., 2012). The protective effect of chronic caffeine administration was accompanied by prevention of weight gain and decreased visceral fat in obese animals; however caffeine also exerted its positive metabolic effects in lean models of insulin resistance and hypertension independently of weight loss (Conde et al., 2012b). A putative mechanism related with blockade of adenosine receptors in the CBs and, therefore, with the inhibition of CB-mediated sympathetic overactivation by chronic caffeine administration has been proposed as a paradigm shift to explain the reduction of insulin resistance, blood pressure and type 2 diabetes risk induced by sustained consumption of this xanthine (Conde et al., 2012b,c; Ribeiro et al., 2013). The translation of these promising results into human medicine, namely through controlled clinical trials is still lacking—but the epidemiological data available strongly indicate that caffeine should integrate a normal healthy diet, and actually contribute to decrease the incidence of type 2 diabetes and obesity in high-risk populations (van Dam and Hu, 2005; Bhupathiraju et al., 2014).

Another way of modulating CB activity would be to directly target its effector, the SNS. The SNS may also represent a putative target to treat metabolic diseases related with insulin resistance, particularly if modulated regionally in classical insulin-target tissues like the skeletal muscle. This pinpoint modulation may be achieved through the use on Bioelectronic Medicines, electronic devices connected to individual peripheral nerve fibers, aiming to correct pathological electrical patterns and restore health (Famm et al., 2013). This new area of therapeutics is emerging right now, with the promise and ambitious goal of modulating specific peripheral nerves. Due to the important role the CBs seem to play in both the metabolic and hemodynamic control, they represent a natural candidate for Bioelectronic Medicines to be tested in a not so distant future.

### Conflict of interest statement

The authors declare that the research was conducted in the absence of any commercial or financial relationships that could be construed as a potential conflict of interest.
